# Normalization of Haemostasis in People with Haemophilia A: Expert Consensus on Unmet Needs and a Framework for Advancing Towards Health Equity

**DOI:** 10.1055/a-2855-7618

**Published:** 2026-05-06

**Authors:** Cedric Hermans, Günter Auerswald, Ruben Berrueco, Gonzalo Calvo, Panagiotis Christoforou, Daniel Anibal-García Diego, Enrico Ferri Grazzi, Paul McLaughlin, Flora Peyvandi, Jan Pucek, Martin Sedmina, Susan Shapiro, Mark W. Skinner

**Affiliations:** 1 Division of Haematology, Cliniques Saint-Luc, Université Catholique de Louvain, Brussels, Belgium; 2 Coagulation Centre, Bremen Central Clinic, GeNo Ltd., Parent-Child-Centre Prof. Hess, Bremen, Germany; 3 Deutsche Hämophiliegesellschaft eV., Hamburg, Germany; 4 Servicio de Hematología Pediátrica, Hospital Sant Joan de Déu de Barcelona, Universitat de Barcelona, Barcelona, Spain; 5 European Association for Clinical Pharmacology and Therapeutics, Barcelona, Spain; 6 Blood Unit and National Reference Center for Congenital Bleeding Disorders, “Laiko” General Hospital, Athens, Greece; 7 Fedhemo (Federación Española de Hemofilia), Madrid, Spain; 8 FedEmo (Federazione delle associazioni Emofilici), Milan, Italy; 9 Katharine Dormandy Haemophilia Centre and Thrombosis Unit, Royal Free London NHS Foundation Trust, London, United Kingdom; 10 Department of Academic Haematology, University College London, London, United Kingdom; 11 Fondazione Istituto di Ricovero e Cura a Carattere Scientifico Ca' Granda Ospedale Maggiore Policlinico, Angelo Bianchi Bonomi Hemophilia and Thrombosis Center, Milan, Italy; 12 Department of Pathophysiology and Transplantation, Università degli Studi di Milano, Milan, Italy; 13 Czech Society of Hemophilia, Prague, Czech Republic; 14 Slovak Hemophilia Society, Bratislava, Slovakia; 15 Oxford Haemophilia and Thrombosis Centre, Oxford University Hospitals, Oxford, United Kingdom; 16 Radcliffe Department of Medicine, Oxford University, Oxford, United Kingdom; 17 Institute for Policy Advancement Ltd, Washington, District of Columbia, United States; 18 Department of Health Research Methods, McMaster University, Hamilton, Ontario, Canada

**Keywords:** consensus, haemophilia A, health equity, treatment outcome

## Abstract

New therapies for haemophilia A have created momentum for enhancing protection against bleeding.

**Objective:**

The ultimate objective is to address remaining unmet needs and promote health equity.

**Methods:**

In this initiative, 14 haemophilia A experts from Europe ( *n* = 13) and North America ( *n* = 1) were involved to identify unmet needs in people with haemophilia A (PwHA) and measures to address these needs (e.g., normalization of factor VIII [FVIII] levels) to facilitate health equity. It combined online workshops, a modified Delphi process to develop consensus statements on remaining unmet medical needs (consensus was defined as ≥70% of panellists who gave a rating of 4 [agree] or 5 [strongly agree] using a 5-point Likert scale), and development of policy recommendations.

**Results:**

Among the panellists who voted, consensus was reached on 25/26 statements, including 13 with 100% agreement. The experts outlined a need to optimize prophylaxis in all eligible PwHA, aiming for high-sustained FVIII levels or normalized haemostasis. The panel also highlighted a need to prevent all bleeds (including microbleeds) that can occur despite prophylaxis, and to address chronic pain which is highly prevalent. Overall, 23 policy recommendations were finalized, which included the wider use of prophylaxis to reduce the burden of disease on PwHA and/or provide normalized haemostasis, the expansion of multidisciplinary teams and the establishment of networks of expertise, and sharing of specialist knowledge.

**Conclusion:**

This initiative lays an ambitious foundation to rigorously evaluate unmet needs in PwHA and the determinants of health equity, offering a comprehensive set of recommendations to overcome barriers and achieve the possibility of normalization of haemostasis.

## Introduction

Routine prophylaxis is advocated by the World Federation of Hemophilia (WFH) guidelines for people with severe or moderate haemophilia with a severe bleeding phenotype, with traditional treatment options including standard and extended half-life factor VIII (FVIII) replacement therapies. 1 The guidelines recommend early prophylaxis with the aim of achieving zero bleeds to improve clinical outcomes such as prevention of joint arthropathy. 1 Historically, prophylaxis aimed to increase FVIII trough levels to >1 IU/dL, but current WFH guidelines now advocate levels of >3 to 5 IU/dL or higher. 1

While current prophylactic therapies have reduced the treatment burden and improved health-related quality of life (HRQoL) compared with previously available therapies, people with haemophilia A (PwHA) remain at risk of (sub)clinical bleeding and joint damage in routine clinical practice. 2 3 4 5 6 The introduction of new therapies in haemophilia A, including a high-sustained activity FVIII replacement therapy, gene therapy, and non-factor therapies, has generated impetus for further improving protection from bleeds and achieving “health equity”.

**Fig. 1:**
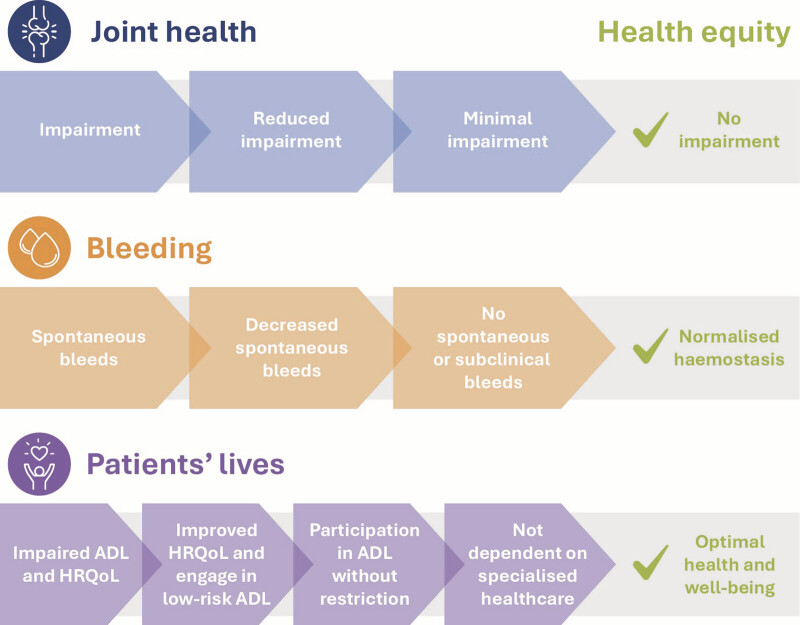
Evolving treatment goals on the path to health equity in haemophilia A. ADL, activities of daily living; HRQoL, health-related quality of life. (Source: Achieving the unimaginable: Health equity in haemophilia, Skinner MW, et al. Copyright © 2020 The Authors. Adapted with permission of John Wiley & Sons Inc.)

The World Health Organization defines health equity as the attainment of full potential for health and well-being for everyone in the absence of unfair, avoidable, or remediable differences among groups of people. 7 In the context of haemophilia A, this refers to an aspiration for PwHA to benefit from the improved pharmacological profiles of new therapies to normalize haemostasis, irrespective of geographic and socio-economic background, aligning the management of haemophilia A with other diseases (e.g., type 2 diabetes). 8 9 The normalization of haemostasis refers to the prevention of all bleeding (including subclinical or microbleeds) and consequently, prevention of pain, avoidance of joint damage (or prevention of further damage), and the achievement of a lifestyle unimpaired by disease complications, irrespective of sex, age, and disease severity ( Fig. 1 ). 9 10 11 12 13 14

In Europe, initiatives such as the European Directorate on standards of care for people with haemophilia 15 and the Interdisciplinary Working Group's principles of comprehensive haemophilia care in Europe 16 provide guidance and recommendations to facilitate haemophilia management, but at present, consensus is lacking on the definition of health equity, its implications for haemophilia A management, and the key unmet needs that may limit the ability to achieve health equity. An expert panel consisting of haemophilia healthcare professionals (HCPs), PwHA, and patient organization representatives was therefore convened to outline the unmet medical needs for PwHA in developed countries, such as those in Europe, and develop consensus statements and policy recommendations to provide a robust framework for initiatives that may help address the gap between the unmet needs and effective delivery of care. Specific objectives were to explore and define health equity for PwHA, and to build consensus among patient, clinical, academic, and industry communities advancing the current care paradigm and enabling sustainable access to life-changing therapies for PwHA.

## Methods

**Fig. 2:**
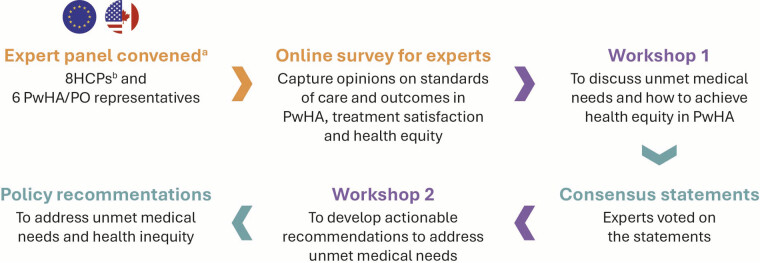
Overview of methods. ^a^ Expert panel members were selected based on their leadership roles in haemophilia A and experience in health policy and advocacy work. The experts were from Belgium, Canada/USA, Czech Republic, Germany, Greece, Italy, Slovakia, Spain, and the UK. ^b^ Seven haematologists and one physiotherapist. HCP, healthcare professional; PO, patient organization; PwHA, people with haemophilia A.

A modified Delphi approach was used to conduct the study, based on two rounds of review of the consensus statements, rather than an unspecified number of reviews until consensus was reached. An overview of the methods is summarized in Fig. 2 .

The expert panel comprised eight haemophilia HCPs and six PwHA/patient organisation representatives from across Europe and North America, selected for their national leadership roles in haemophilia A and their experience in health policy and advocacy. The expert panel was supported by an independent policy and public affairs agency and a medical writing agency throughout the process.

### Development of Consensus Statements

Development of the consensus statements was a four-step process. In the first step, experts participated in online meetings to discuss broad topics - identified through a literature search - related to unmet needs and health inequity in PwHA. Based on these discussions, a survey was developed to evaluate panellists' opinions on standards of care and outcomes in haemophilia A, treatment satisfaction, and the meaning of health equity. The survey, which was completed anonymously online, comprised a series of statements and questions on the following seven topics, identified during the preparatory calls: prophylaxis, pain, outcome measures during prophylaxis, adherence (concordance), multidisciplinary team (MDT) management, knowledge sharing, and economic considerations. Respondents were asked to state their level of agreement (from 1 [strongly disagree] to 5 [strongly agree]) for each question in the survey; they could also include free-text comments regarding each question and provide any additional insights on important points not covered in the survey. Next, panellists attended an online workshop, held during October 2024, to discuss the outputs from the survey. The aims of the workshop were to: explore and define unmet medical needs in haemophilia A that are not covered by standard clinical outcome measurements; and explore and define health equity for PwHA. Following a presentation summarizing the results of the pre-workshop survey, facilitated group discussions were conducted on the key topics of identifying clinical unmet needs in haemophilia A and what defines health equity in haemophilia A. In the final step, during November 2024, a series of consensus statements were developed for each of the seven topics, based on discussions at the workshop and a targeted literature review on each topic, focusing on articles published in the previous 5 to 10 years. The first draft statements underwent two rounds of review by the expert panel and were rephrased based on the feedback provided after each review. Panellists then accessed an online, anonymized voting platform (Microsoft Forms) to vote on the final statements, using a 5-point Likert scale (where 1 = strongly disagree, 2 = somewhat disagree, 3 = neither agree nor disagree, 4 = somewhat agree, and 5 = strongly agree). Consensus was defined during the second workshop as ≥70% of the panellists scoring 4 or 5; this threshold for consensus was consistent with other consensus-building studies in haemophilia. 17 18

### Development of Proposed Policy Recommendations

A second online workshop (January 2025) was held to discuss a set of actionable, evidence-based and aspirational policy recommendations which aim to address the unmet needs in the seven key areas: (1) reaching non-haemophilia range FVIII levels through prophylaxis; (2) rethinking outcome measures during prophylaxis; (3) pain management; (4) rethinking adherence and shared decision-making; (5) bringing MDTs into a new ‘haemophilia’ era; (6) promoting health literacy and continued medical training; and (7) the value of haemophilia treatment and care. The first draft of the policy recommendations was developed by the independent consultancy agency. This draft was based on an anonymous pre-workshop online survey and feedback from Workshop 1, incorporating multiple perspectives from the experts. The first draft of the policy recommendations was then discussed with the expert panel during Workshop 2, followed by two rounds of review (including online and email feedback) and approval.

## Results

**Fig. 3:**
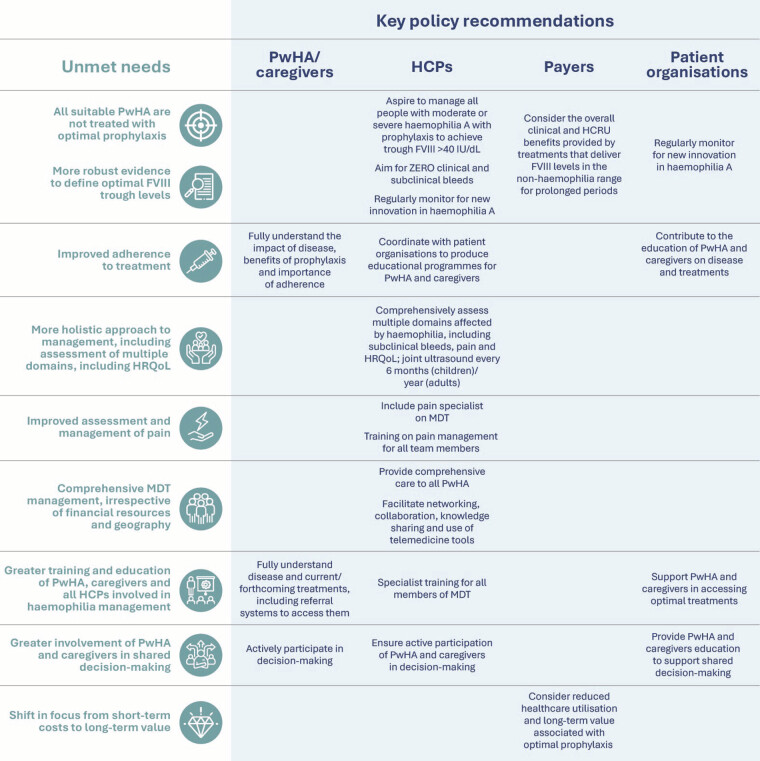
Unmet needs in people with haemophilia A (PwHA) and key policy recommendations for achieving evolving health equity in haemophilia A. FVIII, factor VIII; HCP, healthcare professional; HCRU, healthcare resource utilization; HRQoL, health-related quality of life; MDT, multidisciplinary team.

A summary of the unmet needs identified in PwHA and key policy recommendations to address these and facilitate the move towards health equity are shown in Fig. 3 . A key unmet need was that not all eligible PwHA receive the optimal level of prophylaxis to achieve the best possible outcomes, based on achieving optimal FVIII levels, preventing clinical and subclinical bleeds, preservation of joint health, improvements in pain, activities of daily living and HRQoL, and minimal burden of treatment. Other key unmet needs include chronic pain which is highly prevalent and requires effective management, including pain specialists in the MDT; and not all eligible PwHA receive comprehensive care via an MDT, which is critical for optimizing outcomes.

### Consensus Statements

All 11 panellists voted on 21 of the 26 statements; one panellist did not vote on three of the statements and two panellists did not vote on one statement each (the reason[s] for missing votes could not be established). The consensus threshold (≥70% of respondents ‘strongly agreed’ or ‘somewhat agreed’) was reached for 25/26 statements, including 13 with 100% agreement ( Table 1 ). Additional key points associated with the consensus statements from the expert panel are summarized below.

**Table 1:** Consensus statements and levels of agreement

Statement	Agreement a
***Prophylaxis***	
Spontaneous joint bleeds and subclinical bleeds (also referred to as microbleeds) can occur even with prophylaxis, which increases the risk of long-term joint damage in PwHA	100%
The choice of prophylactic treatment will depend on the individual's clinical condition and preferences, the safety profile of each therapy, and its accessibility and affordability, but clinicians should strive to achieve optimal prophylaxis with the aim for each PwHA to achieve health equity and consequently attain a life undeterred by disease complications	100%
High-sustained FVIII products used once weekly can achieve FVIII levels in the non-haemophilia range for prolonged periods, while extended half-life products will need to be used every 1 to 2 days (associated with increased burden and constitutes off-label use) to achieve the same level of protection. Barriers to routinely providing non-haemophilia range levels of protection may include costs and policy restrictions	90% b
All PwHA should receive effective prophylaxis to achieve FVIII levels or equivalent haemostatic activity high enough to prevent, as a minimum, all spontaneous bleeds; on–demand treatment should not be used	82%
Treatment options which can achieve FVIII levels in the non-haemophilia range for prolonged periods are expected to be available for PwHA in Europe in the near future	82%
Heterogeneity in the availability of effective therapy between countries and even regions within the same country is one of several barriers to providing prophylaxis to all PwHA	73%
***Outcome measures during prophylaxis***	
Haemophilia treatment goals need to be re-defined beyond annualized bleeding rates and should consider chronic pain, joint damage, functioning, social participation, quality of life, and, ultimately, health equity relative to the general population	100%
There is an urgent need to establish consensus among medical professionals, patients, and policy and decision makers on how to best assess the overall impact of care for PwHA, particularly with respect to PROMs and PREMs	91%
***Pain***	
In some PwHA, pain is a consequence of irreversible arthropathy and can only be minimized by appropriate approaches such as high FVIII levels and MDT care. However, the perception that pain is an unavoidable consequence of the disorder should be challenged, as early optimized prophylaxis can prevent bleeds, thus preventing the development of arthropathy-associated pain	100% b
There is a need for more education and training on optimal pain management amongst haemophilia healthcare teams and greater involvement of pain specialists, ideally as part of the MDT responsible for managing PwHA	91%
Chronic pain is highly prevalent in PwHA, and its effective management is a major unmet need	82%
Pain is an indicator of poor joint health and plausibly an indicator of prior or current suboptimal haemostasis with insufficient FVIII levels	73%
Some people with severe or non-severe haemophilia A may experience haemophilia-related pain. The use of painkillers in these patients adds to the burden of disease, and in serious cases may increase the risk of cardiovascular disease or dependence/addiction	60% b
***Adherence***	
Non-adherence in PwHA may be attributed to the burden of treatment, venous access issues, a lack of knowledge amongst PwHA on the benefits of prophylaxis, and accessibility issues to different treatment options and services	100%
Advances in treatment options for PwHA are expected to alleviate the burden of treatment; these could benefit young adults and adolescents by providing greater convenience to fit with their lifestyle, as well as for older age groups by providing therapies that are simpler to administer and require less frequent injection	100% b
There is a need to standardize the assessment of adherence, which may vary between countries and regions	82%
***MDT management***	
Comprehensive care by an MDT of specialists is critical for optimizing the management of PwHA and achieving health equity. However, there is a need to improve access to comprehensive care services as this is highly variable among different regions and/or countries	100%
PwHA have an improved life expectancy but are more likely to experience comorbid conditions during their lifetime; this means that MDTs may have to be extended to include, for example, geriatricians and cardiologists who have an understanding of haemophilia A	100%
***Knowledge sharing***	
There is a need for ongoing education for PwHA on the disease and its management to facilitate shared decision-making with HCPs; patient associations and haemophilia societies can play a key role in educating PwHA on general aspects of haemophilia A management	100%
Caregivers of PwHA also require education to address potential uncertainties surrounding the condition and its treatment	100%
PwHA, caregivers, and all HCPs involved in managing PwHA need to be empowered to better understand the national healthcare structures and decisions that limit access to innovative therapies and care through sharing of knowledge	100%
There is a need for greater awareness of and education around the healthcare needs for females with haemophilia A, including prolonged bleeding during menstruation or after an accident or surgery, and uncertainties relating to pregnancy and delivery	100%
Achieving health equity could be facilitated by greater networking and knowledge-sharing between national health authorities, development of patient-centric guidelines, and better alignment between guidelines and policy implementation	80% b
***Economic considerations***	
There is a need for ongoing dialogue with payers to drive policy change, which recognizes the value of prophylactic treatments capable of achieving non-haemophilia FVIII target levels	100%
Haemophilia A is a chronic condition that requires lifelong treatment. Approximately 90% of the costs of managing PwHA are attributed to the cost of therapies. However, there is a need to shift the focus of optimal prophylactic treatments from acquisition costs to the overall long-term economic value provided	91%
Additional studies are required to better estimate the overall impact of prophylaxis for PwHA with respect to functional outcomes (e.g., ability to attend school and work), current and future healthcare usage/budgets, and quality of life	82%

Abbreviations: FVIII, factor VIII; HCP, healthcare professional; MDT, multidisciplinary team; PREM, patient–reported experience measure; PROM, patient–reported outcome measure; PwHA, person/people with haemophilia A.

Note: Shaded cells represent statements for which consensus (agreement ≥70%) was not reached.

a Proportion of participants scoring 4 or 5, where 4 = somewhat agree and 5 = strongly agree.

b *n* = 10 respondents (all other statements voted on by all 11 participants).

#### Prophylaxis

Consensus was reached on all six statements on prophylaxis. It was agreed that PwHA should be managed with prophylaxis rather than on-demand treatment, but more research is needed to determine the potential benefits of prophylaxis in those with moderate haemophilia A and a non-severe phenotype, and in those with mild haemophilia. More research is also needed to determine the optimal target FVIII trough levels or ‘area-under-the-curve (AUC)’ considering potential implications for treatment burden, but a key aspiration is to aim for FVIII levels within the normal range (normalization of haemostasis).

#### Outcome Measures During Prophylaxis

Consensus was reached on both statements. The need to proactively evaluate outcomes across multiple domains was emphasized, including bleeds, joint damage, activities of daily living, HRQoL, and treatment burden. Numerous tools are available for measuring functioning and HRQoL. It would be useful to have a general tool that captures all clinically relevant factors for PwHA. Also highlighted was a need to challenge the misconception amongst some clinicians that a small number of bleeds per year is acceptable, without considering the long-term implications on joints and HRQoL.

#### Pain

Consensus was reached on four of the five statements; 60% of respondents agreed with the fifth statement. Panellists highlighted that use of medications to treat pain should be individualized and carefully managed, and additional studies are required in PwHA. The experts discussed that some of the more recent treatment advances may help to ameliorate pain, and a multidisciplinary approach may support good management of pain. They highlighted the importance of including a pain specialist within an extended MDT and the need for education on pain management for those managing PwHA.

#### Adherence (Concordance)

Consensus was reached on all three statements. Adherence to prophylaxis was highlighted as a key factor in ensuring optimal outcomes in PwHA. It was noted that reasons for non-adherence are multifactorial, including poor health literacy, forgetting or reluctance to take medication, and/or the burden of treatment.

#### Multidisciplinary Team (MDT) Management

Consensus was reached on both statements. It was emphasized that access to an MDT, irrespective of geographical location, is key to ensuring optimal outcomes, but such access is highly variable among PwHA. It was also highlighted that the increase in life expectancy as a result of major advances in haemophilia management may require expansion of the MDT to include other specialities such as geriatricians, cardiologists, endocrinologists, and oncologists.

#### Knowledge Sharing

Consensus was reached on all five statements. The need for sharing of knowledge and experience between larger comprehensive care centres and smaller haemophilia treatment centres was noted. This includes sharing information about available treatments and how to integrate these into the everyday lives of PwHA. Education for PwHA and their caregivers is also needed to ensure they are fully involved in the shared decision-making process; national or international patient organizations/haemophilia societies could provide a supportive role.

#### Economic Considerations

Consensus was reached on all three statements. It was highlighted that addressing lack of reimbursement of more recently approved therapies in some patient subgroups/some countries could facilitate health equity. It was also noted that payers and physicians should be aligned on the need to improve therapeutic standards; in addition, decision makers and payers should give full consideration to long-term economic and equity benefits of prophylaxis and novel therapies when assessing short-term acquisition costs.

### Policy Recommendations to Address Unmet Needs and Achieve Health Equity for PwHA

A total of 23 policy recommendations were developed by the expert panel ( Supplementary Table S1 ). The policy recommendations include proposals regarding the effective use of prophylaxis to achieve normalized haemostasis, the expansion of MDTs, widening comprehensive care services, and increased collaboration between HCPs and patient organizations.

## Discussion

The aims of this initiative were to develop consensus statements and actionable policy recommendations to support a new goal of establishing health equity for all PwHA with respect to normalization of haemostasis. A key aspiration highlighted by the panellists was to extend prophylaxis to all people with severe or moderate haemophilia A, not only those with a severe phenotype. 1

Current WFH guidelines state that most clinicians support aiming for FVIII trough levels of ≥3 to 5 IU/dL. 1 However, studies suggest PwHA are not fully protected from (sub)clinical bleeds and/or joint damage despite prophylaxis at such levels. 6 19 20 21 22 Other studies (modelling of both retrospective and prospective data) have demonstrated that every 1 IU/dL increase in baseline FVIII levels is associated with decreased bleeding frequency in PwHA, and FVIII activity levels of 15 to 50 IU/dL are associated with a near-zero bleed rate. 14 23 24 25 26 27 28 29 30 Once-weekly prophylaxis with efanesoctocog alfa, which maintains FVIII levels at >40 IU/dL for the first 4 days and >15 IU/dL on day 7 (based on subgroup pharmacokinetic assessments), was associated with a median annualized bleeding rate (ABR) of zero (interquartile range, 0–1.04) in adults/adolescents with severe haemophilia A. 31 Even though it is difficult to quantify, the non-factor therapy, emicizumab, is estimated to have haemostatic activity equivalent to 10 to 20 IU/dL 32 and therefore may not have the potential to normalize haemostasis.

A key aspiration identified by the expert panellists is aiming towards normalized haemostasis by sustaining FVIII levels in the near-normal/normal range (trough levels >40 IU/dL); this is proposed as a mechanism to achieve health equity. 9 10 12 13 33 While results are promising, additional long-term studies are required to evaluate outcomes in PwHA who sustain FVIII levels at >40 IU/dL. The AUC, which best measures exposure to the treatment, can also provide insights on the therapeutic window and potential bleeding risk, including (in theory) subclinical bleeds. 34 It is also acknowledged that measures other than FVIII levels may be needed to assess the optimal level of haemostasis achieved with bispecific antibodies like emicizumab and haemostasis-rebalancing agents that increase thrombin formation. 35

High-sustained activity FVIII products and gene therapy can achieve normalization of haemostasis for prolonged periods in a proportion of PwHA. 13 36 These treatments are available for severe haemophilia in some European countries and are expected to become more widely available in the near future. It is important that clinicians and PwHA have access to these treatments, that access is extended to those with less severe haemophilia, and that haemophilia guidelines are updated in a timely manner to reflect the benefits of these treatments. Of note, the WFH published guidelines for gene therapy in late 2025, 37 but the guidelines remain to be updated to reflect high-sustained activity FVIII products and haemostasis-rebalancing agents. The panellists also recommended that patient organizations and clinicians should regularly monitor for therapeutic advances, and report their real-world effectiveness and safety.

The panel recommended that the efficacy of prophylaxis is evaluated proactively across multiple domains. Traditionally, treatment decisions in PwHA have been based mainly on ABRs. However, studies show that even a single bleed can cause irreversible joint damage 38 39 and subclinical bleeds can cause joint damage, even if prophylaxis prevents clinically evident bleeds. 3 4 38 40 41 Regular assessment by ultrasound appears to assist in early diagnosis of joint damage, facilitating actions that may prevent progression to haemophilic arthropathy. 42 43 The panel suggested that ideally, ultrasound exams of all major joints should be conducted every 6 months in children (<18 years) and at least once a year for adults, while acknowledging that availability of equipment and trained staff may vary and there may be time burden for PwHA for attending appointments. Ultrasound can also be used to monitor response to treatment, 42 but further investigation of the benefits of regular ultrasound on long-term outcomes is needed, including the relative benefits for children versus adults and those with or without existing arthropathy.

The importance of capturing subjective patient-reported outcomes (PROs) in PwHA was also highlighted by the panel. At present, multiple PRO tools are available, 44 which can make it difficult to compare the results from different research studies and can increase the time (beyond regular clinical assessments) of clinical appointments. A pilot study of the PROBE project (a patient-led initiative to develop a single questionnaire of haemophilia-related health problems and HRQoL) reported that all people with haemophilia (PwH) ( *N* = 656) completed the questionnaire within 30 minutes and 71% completed it within 15 minutes. 45 In the context of HRQoL, it is important to note a phenomenon called the ‘disability paradox’, whereby PwHA simply accept the impact of their condition, adapting to their level of disability and reporting better than expected HRQoL relative to the general population, thus underestimating the impact of haemophilia A. 46

The expert panel agreed that effective management of chronic pain, which is highly prevalent in PwHA, remains unsatisfactory due to limited therapeutic approaches, a lack of understanding on underlying pain mechanisms in haemophilia (and whether these differ from the general population) and a lack of specific evidence on pain management strategies in PwHA. Although pain in PwHA can be managed using a variety of strategies, 47 48 the United Kingdom 49 and International Society on Thrombosis and Haemostasis guidelines 50 do not provide recommendations on the management of pain, while the WFH guidelines provide some guidance (stepwise approach according to severity). 1 When approaching pain management, it may be helpful to stratify patients according to prior arthropathies and/or joint surgery. A group of haemophilia experts examining pain management concluded that the effective management of pain is influenced by factors such as individual knowledge/skill and wider geographical determinants. 51

The experts identified a need for training and education for clinicians, PwHA, and caregivers on the role of effective prophylaxis to prevent long-term joint damage and chronic pain, and on pain management strategies. The panel also highlighted that the perception that pain is unavoidable should be challenged, highlighting that although this may be the case for those with pre-existing haemophilic arthropathy, early optimized prophylaxis can prevent bleeds, thus preventing the development of arthropathy-associated pain. 31 47 52 53 Some studies have reported prophylaxis with current therapies can improve pain intensity and pain-related HRQoL, 31 52 but additional studies are required to confirm an association between preventing bleeds and avoiding pain.

The panel noted that comprehensive care via MDTs is critical for optimizing outcomes in PwHA, including the management of acute and chronic pain, a view that is endorsed by the National Bleeding Disorders Foundation guidelines. 54 As such, the MDT should, ideally, have access to pain specialists, physiotherapists, rheumatologists, and orthopaedic surgeons. 55 56 However, a study conducted in Italy reported clinicians did not unanimously agree with including pain specialists within comprehensive care teams; this may reflect the lack of availability of pain specialists at the centres involved and that pain management was handled by a haemophilia specialist in >90% of cases. 57

Education of PwH and shared decision-making were also recognized by the expert panel as integral to effective management of haemophilia A, in order to improve treatment adherence 58 59 and produce better outcomes. 60 61 In the spirit of shared decision-making, the term ‘concordance’ may be preferred to ‘adherence’ as it emphasizes a shared understanding and agreement between the patient and healthcare provider about the treatment plan. 62 The WFH has developed a shared decision-making tool that provides information on the individual's life goals, impact of haemophilia, treatment preferences, and available therapies. 63 As well as educating PwHA, there is a need for ongoing education of HCPs involved with managing PwHA via sharing of knowledge and experience. National and international networks of haemophilia centres, including the EUHANET project, 64 65 could be established/expanded to facilitate knowledge sharing, training, and education.

Finally, and importantly, the panel members agreed on the need to adopt a holistic approach to haemophilia-related costs (i.e., shift the focus from acquisition costs to overall long-term economic value) and a requirement for more data on the economic impact of sub-optimal outcomes and reduced productivity and well-being. Real-world studies show that drug acquisition costs account for most of the costs associated with managing haemophilia, while non–drug-related medical costs (e.g., those associated with bleeding and problem joints) and societal costs (e.g., lost productivity) are also key cost factors; direct and indirect costs appear to increase with worsening clinical status. 66 67 68 69 Also, in resource-constrained countries, the WFH guidelines specify that prophylaxis rather than on-demand therapy should be used, acknowledging that less intensive regimens may be used if cost is an issue, but highlighting that in all countries, the aim is to achieve zero bleeds. 1

Limitations of the current study include the relatively small expert panel and relatively small number of countries represented. However, the views and opinions expressed by the panel were broadly consistent with the published literature. Use of a modified Delphi process may also be seen as a limitation, but the consensus threshold was reached for all but one of the statements at the first round of voting, so a second round of voting was not necessary. The findings from this study may be broadly applicable for other resourced countries, but further studies are required to help address health equity in lower-middle income countries or other regions. A strength of the study was that it included perspectives from clinicians, allied HCPs, and PwHA and their representatives from Europe and North America. Another strength was that it captured a wide range of issues and possible solutions that impact on unmet needs and health equity in PwHA.

## Conclusion

Despite significant advances in the management of haemophilia A in recent decades, PwHA still face a number of unmet needs and much remains to be done if we are to achieve health equity, irrespective of financial resources, healthcare provision, or geographical location. This consensus initiative with leading international experts identified a number of factors that will drive health equity in PwHA, including the aspiration to sustain FVIII levels in the normal range for those with moderate/severe haemophilia on prophylaxis, challenging the misconception that a small number of bleeds per year is acceptable, and minimizing or preventing chronic pain with the use of early, optimal prophylaxis and an extended MDT. By addressing these, the aim is to improve outcomes for PwHA in developed countries, including those in Europe, by utilizing current therapeutic options and increasing collaboration and education. This initiative lays an aspirational foundation to rigorously evaluate the remaining unmet needs in PwHA and the determinants of health equity, offering a comprehensive set of recommendations to surmount barriers and achieve the possible normalization of haemostasis, once considered ‘the unimaginable’.

## Notes

**What Is Known About This Topic?**

- Despite routine prophylaxis, PwHA continue to experience subclinical bleeding, chronic pain, and impaired health-related quality of life, indicating residual unmet clinical need.
- Disparities in access to optimal prophylaxis, comprehensive multidisciplinary care, and patient education across treatment centres/regions limit the achievement of health equity.
- Achieving FVIII levels within the non-haemophilic range is a clinically meaningful target that may help reduce outcome disparities and advance haemophilia A care.

**What Does This Paper Add?**

- This paper presents an expert consensus defining normalization of haemostasis – characterized by achieving near-normal FVIII levels (>40 IU/dL) – as an aspirational treatment goal in haemophilia A and a proposed mechanism to prevent bleeding, minimize disease burden and achieve health equity.
- It identifies unmet needs across seven key domains and outlines 23 actionable policy-oriented recommendations aimed at advancing health equity.
- The study proposes a structured framework linking clinical outcomes, patient-centred outcomes and health-policy factors, with emphasis on multidisciplinary care, patient education, and long-term value.
