# Supplementary material for: Normalization of Haemostasis in People with Haemophilia A: Expert Consensus on Unmet Needs and a Framework for Advancing Towards Health Equity
Source: TH Open. 2026 May 6;10:a28557618. doi: 10.1055/a-2855-7618 (PMC13334954; doi:10.1055/a-2855-7618)
Supplement: Supplementary file 1 — Supplementary Material [file 10-1055-a-2855-7618_28686033.pdf]

Normalisation of Haemostasis in People with Haemophilia A: Expert Consensus on Unmet Needs and a Framework for Advancing Towards Health Equity

Supplementary Materials

Table S1 Policy recommendations

1. Policy recommendations for prophylaxis
a. All people with moderate or severe haemophilia A, and/or with a severe clinical phenotype,[a] should receive prophylaxis. Compared with standard and extended half-life factor VIII (FVIII) therapies, advanced treatments for haemophilia A including non-factor therapies, gene therapy and high-sustained FVIII replacement therapy can reduce burden and/or provide normalisation of haemostasis (i.e. FVIII levels >40 IU/dL), thereby improving protection against bleeds and joint damage.[1,2] Other proposed benefits associated with sustained haemostatic control include improved health-related quality of life (HRQoL), reduced pain and improved participation in physical activities.[1–3]
b. While prophylaxis with non-factor therapies and high-sustained FVIII replacement therapy can provide effective protection from bleeding and help to preserve joint health,[3,4] it is important that prophylaxis for people with haemophilia A (PwHA) should be personalised to their needs, including spontaneous bleeds and monitored to optimise clinical outcomes.
c. Treatment options that can achieve normalised haemostasis (FVIII >40 IU/dL) for prolonged periods are already available or are expected to be available for PwHA in Europe soon. Unlocking access to innovation can bring significant improvements in HRQoL by tapping into the potential for higher protection for PwHA with different severities or bleeding phenotypes, ultimately reducing the gap in health outcomes for different sub-groups of PwHA and bringing FVIII levels closer to those in their non-haemophilia peers.
d. Clinical guidelines that can support the achievement of health equity for PwHA should be informed by existing and forthcoming clinical evidence in a timely manner; for example, current treatment guidelines do not always foresee prophylaxis regimes for people with moderate forms of haemophilia A, and as a

TH Open | DOI 10.1055/a-2855-7618 | © The Author(s).

	result, these PwHA often experience outcomes inferior to people with severe haemophilia A, who are on prophylaxis regimes. In addition, high-sustained FVIII activity levels need to be ensured throughout an individual's lifespan if chronic joint damage and the associated long-term deterioration of physical function and HRQoL are to be effectively prevented.
e.	Ultrasound exams of all major joints should be conducted every 6 months in children (<18 years) and at least once a year for adults – especially those with severe haemophilia A and/or a severe clinical phenotype – to monitor and prevent (further) joint damage. Physicians should adapt treatment and care (e.g. physiotherapy) according to the evolution of joint damage and the needs of PwHA. In addition, ultrasound findings should inform more personalised treatments and/or medical advice for PwHA.
**2. Policy recommendations for pain management**	
a.	All PwHA, irrespective of their disease severity, age and gender, should be regularly assessed for chronic haemophilia-related pain.
b.	All PwHA suffering from chronic pain should be proactively offered a multidisciplinary pain management plan composed of both pharmacological and non-pharmacological interventions. Access to physiotherapy and psychosocial support should also be featured in multidisciplinary pain management plans.
c.	Multidisciplinary pain management, including practical examples on developing a pain management plan, guidance on medicines use (both haemophilia A treatments and pain killers) and the management of side effects and addiction, should be developed and included in existing clinical guidelines. Guidelines for pain management should also tailor treatment to different age groups, genders and the specific pain issues pertaining to these different categories.
d.	Existing guidelines on the comprehensive care services offered in European haemophilia centres, such as those used by the European Association for Haemophilia and Allied Disorders (EAHAD) to certify European treatment centres,[5] should be updated to reflect the need for inclusion of pain management as a specialty within the centre.
e.	Clinicians, PwHA and caregivers should regularly receive training and educational activities on pain management through both pharmacological and non-pharmacological interventions. This training highlights the scope for greater cooperation between patient organisations and haemophilia treatment centres.

TH Open | DOI 10.1055/a-2855-7618 | © The Author(s).

3. Policy recommendations for ensuring adherence and shared decision-making in haemophilia A
a. Shared decision-making should be at the heart of haemophilia A treatment and result in treatment protocols and objectives collaboratively defined by healthcare professionals, PwHA, and, where applicable, caregivers.
b. There should be a corresponding shift in how 'adherence' is perceived. Emphasis should be placed on healthcare professionals ensuring that PwHA and their caregivers are included in the clinical decision-making process. PwHA and caregivers should not bear the full responsibility of meeting treatment goals if these are imposed without sufficient consultation. Education for PwHA/caregivers can be especially important in relation to adherence for the paediatric and adolescent populations.
**4. Policy recommendations for multidisciplinary treatment in a new haemophilia 'era'**
a. Specialist multidisciplinary health services play a key role in improving health outcomes in PwHA. These teams need to be continuously trained to keep up with developments in science and medicine, as well as with the evolving needs of the community of PwHA, e.g. by broadening the scope of health services to include women with haemophilia (even those with mild disease but severe phenotype resulting in life-impairing symptoms) or an ageing population requiring specialist healthcare providers such as cardiologists, geriatricians or neurologists.
b. Access to specialist MDT services is highly dependent on the geographical location of haemophilia centres. To reduce access gaps, MDTs should consider increasing the use of telemedicine tools and planning MDT services around visits to the haematologist.
c. Comprehensive MDT services are already considered an integral part of haemophilia treatment, but further efforts are required to demonstrate their impact on PwHA, quality of life and cost-effectiveness, with a view of informing health budget allocations for the MDT.
d. Access to specialist MDTs is generally dependent on the availability of specialist workforce. This issue is part of a more significant structural issue in Europe on a shortage of health workforce, and the haemophilia community should contribute to this broader debate by demanding further training and job

TH Open | DOI 10.1055/a-2855-7618 | © The Author(s).

attractiveness for young haematologists and allied healthcare service graduates, such as physiotherapists.
e. Access to specialist MDTs can only occur when a referral system facilitates transition from primary care into secondary care. PwHA and clinicians should develop advocacy efforts at the national level to ensure that care pathways are put in place according to local requirements and healthcare system contexts.
**5. Policy recommendations for promoting health literacy and continued medical training**
a. Considering the evolving treatment landscape and advances in science and medicine, healthcare professionals, PwHA, and their caregivers require ongoing education on research developments into their disease and its clinical management. Health literacy is the basis for shared decision-making, and the haemophilia A community needs to be accurately informed on advances in haemophilia treatments, as well as the broader health environment in which treatment services are delivered.
b. Patient organisations and clinicians should regularly monitor incoming innovation in haemophilia A treatments and inform, at a minimum, on a quarterly basis the community, to ensure that all PwHA benefit from the highest level of protection.[6]
c. Patient organisations, together with healthcare professionals, should develop educational programmes and information material adapted to different life stages of PwHA and caregivers as well as to different haemophilia populations.
d. Continuous education is also required for healthcare professionals who regularly see those with haemophilia as well as for those who may not be regularly in contact with PwHA – such as emergency medicine specialists – and for which a basic knowledge of haemophilia is crucial to ensure optimal healthcare service delivery.
e. Healthcare professionals should promote haemophilia treatment centres to organise around regional, national and international networks of expertise and to share specialist knowledge. Knowledge-sharing can promote continuous medical development, collaboration, and, potentially, research into clinical practice in haemophilia treatment and care.
**6. Policy recommendations for value assessments of treatment and care**

TH Open | DOI 10.1055/a-2855-7618 | © The Author(s).

a. There needs to be a shift in the long-term value assessment for haemophilia treatment and care, with developments in evidence generation necessary to support changes in treatment evaluations. A comprehensive approach to data generation on the real-world impact of haemophilia A treatments and management on the lives of PwHA – beyond clinical endpoints – and their uptake in decision-making can broaden treatment options and targets in clinical practice through informed evaluations of innovation, ultimately driving forward optimal health outcomes for PwHA. Long-term data regarding treatments' impact on joint health, bleed control and mental wellbeing, social engagement and work/educational productivity level need to be consistently taken up in payer decision-making.
b. Such studies could better equip PwHA and clinicians for broader discussions on sustainable economic investments in haemophilia health services and treatment.

Abbreviations: FVIII, factor VIII; HRQoL, health-related quality of life; MDT, multidisciplinary team; PwHA, people with haemophilia A.

[a]The definition of severe clinical phenotypes should be gender sensitive to include, for example, more severe menstrual bleeding in women living with haemophilia A.

TH Open | DOI 10.1055/a-2855-7618 | © The Author(s).

Thieme

References

1 Gollard R. The benefits of sustaining high factor VIII levels in people with hemophilia A. Am J Manag Care 2025;31(2 Suppl):S15–S22

2 Carcao M, Selvaratnam V, Blatny J. How much prophylaxis is enough in haemophilia? Haemophilia 2024;30(Suppl 3):86–94

3 Von Drygalski A, Chowdary P, Kulkarni R, et al. Efanesoctocog alfa prophylaxis for patients with severe hemophilia A. New Engl J Med 2023;388(04):310–318

4 Kiialainen A, Niggli M, Kempton CL, et al. Effect of emicizumab prophylaxis on bone and joint health markers in people with haemophilia A without factor VIII inhibitors in the HAVEN 3 study. Haemophilia 2022;28(06):1033–1043

5 European Haemophilia Centres Accreditation. Accreditation of European Haemophilia Centres. Accessed June 2025. https://www.eahad.org/eahad-projects/european-haemophilia-centres-accreditation/

6 World Federation of Hemophilia. WFH guidelines for NMO capacity development 2022. Accessed June 2025. https://www1.wfh.org/publications/files/pdf-2295.pdf

TH Open | DOI 10.1055/a-2855-7618 | © The Author(s).
